# Design of a Convolutional Neural Network as a Deep Learning Tool for the Automatic Classification of Small-Bowel Cleansing in Capsule Endoscopy

**DOI:** 10.3390/medicina59040810

**Published:** 2023-04-21

**Authors:** Tiago Ribeiro, Miguel José Mascarenhas Saraiva, João Afonso, Pedro Cardoso, Francisco Mendes, Miguel Martins, Ana Patrícia Andrade, Hélder Cardoso, Miguel Mascarenhas Saraiva, João Ferreira, Guilherme Macedo

**Affiliations:** 1 Department of Gasteroenterology, São João University Hospital, Alameda Professor Hernâni Monteiro, 4200-427 Porto, Portugal; 2 Gastroenterology and Hepatology, WGO Gastroenterology and Hepatology Training Centre, 4050-345 Porto, Portugal; 3 Faculty of Medicine, University of Porto, 4200-319 Porto, Portugal; 4 Endoscopy and Digestive Motility Laboratory, ManopH, 4000-432 Porto, Portugal; 5 Department of Mechanical Engineering, Faculty of Engineering, University of Porto, Rua Dr. Roberto Frias, 4200-465 Porto, Portugal; 6 INEGI—Institute of Science and Innovation in Mechanical and Industrial Engineering, 4200-465 Porto, Portugal

**Keywords:** capsule endoscopy, artificial intelligence, bowel preparation, deep learning

## Abstract

*Background and objectives*: Capsule endoscopy (CE) is a non-invasive method to inspect the small bowel that, like other enteroscopy methods, requires adequate small-bowel cleansing to obtain conclusive results. Artificial intelligence (AI) algorithms have been seen to offer important benefits in the field of medical imaging over recent years, particularly through the adaptation of convolutional neural networks (CNNs) to achieve more efficient image analysis. Here, we aimed to develop a deep learning model that uses a CNN to automatically classify the quality of intestinal preparation in CE. *Methods*: A CNN was designed based on 12,950 CE images obtained at two clinical centers in Porto (Portugal). The quality of the intestinal preparation was classified for each image as: excellent, ≥90% of the image surface with visible mucosa; satisfactory, 50–90% of the mucosa visible; and unsatisfactory, <50% of the mucosa visible. The total set of images was divided in an 80:20 ratio to establish training and validation datasets, respectively. The CNN prediction was compared with the classification established by consensus of a group of three experts in CE, currently considered the gold standard to evaluate cleanliness. Subsequently, how the CNN performed in diagnostic terms was evaluated using an independent validation dataset. *Results*: Among the images obtained, 3633 were designated as unsatisfactory preparation, 6005 satisfactory preparation, and 3312 with excellent preparation. When differentiating the classes of small-bowel preparation, the algorithm developed here achieved an overall accuracy of 92.1%, with a sensitivity of 88.4%, a specificity of 93.6%, a positive predictive value of 88.5%, and a negative predictive value of 93.4%. The area under the curve for the detection of excellent, satisfactory, and unsatisfactory classes was 0.98, 0.95, and 0.99, respectively. *Conclusions*: A CNN-based tool was developed to automatically classify small-bowel preparation for CE, and it was seen to accurately classify intestinal preparation for CE. The development of such a system could enhance the reproducibility of the scales used for such purposes.

## 1. Introduction

Capsule endoscopy (CE) is a minimally invasive enteroscopy procedure that can be used to examine the small-bowel mucosa and study conditions affecting the small intestine, such as obscure gastrointestinal bleeding (OGIB), small-bowel tumors, Crohn’s disease, polyposis syndromes and celiac disease [1,2,3,4,5]. The quality of mucosal visualization and, hence, the diagnostic potential of CE are highly dependent on the cleanliness of the digestive tract and may be hindered by air bubbles or the presence of bile and intestinal debris. Adequate bowel preparation underlies the cleansing of the small intestine and the optimal assessment of the entire mucosa, facilitating conclusive CE examinations [6,7,8,9,10]. While there are international guidelines on small-bowel CE (SB-CE) preparation to ensure better mucosal visualization [11,12,13], there is still no consensus regarding the optimal small-bowel preparation to improve diagnostic yield [14]. This is in part due to the lack of a reliable tool to assess the quality of mucosa visualization prior to performing a CE. A range of qualitative and semi-quantitative cleanliness scales can be applied to SB-CE, with quite different technical characteristics [15], although the reproducibility of these is generally poor, as reflected by the high intra- and inter-observer variability reported [16,17,18]. A single CE video examination produces as many as 50,000 images, which require considerable time and effort to review [11,19]: at least 30–120 min of dedication by a gastroenterologist [20]. Moreover, small-bowel abnormalities or lesions might only be visible in relatively few frames, such that manual reading is associated with an inherent risk of overlooking clinically important information and it is subject to the limitations of the reader’s ability and concentration [19].

To overcome these drawbacks, AI tools have emerged in recent years that can help optimize the reading process, assisting specialists in the identification of areas of interest and of suspicious abnormalities. For example, AI algorithms have been designed with a view to detect lesions automatically, to classify bowel cleanliness, and to automatically compartmentalize the small bowel in SB-CE sequences [21]. Convolutional neural networks (CNNs) are AI-based multi-layered deep learning algorithms that are particularly well suited to automatic image analysis and pattern recognition [22]. Indeed, the application of CNN-based tools to endoscopy imaging, and specifically to CE examinations, has achieved excellent performance in detecting and classifying a range of diseases [23,24]. Thus, the integration of such AI-based technology into CE protocols may improve the diagnostic accuracy of these procedures and reduce the burden on gastroenterologists, reducing the time spent reading the images, as well as the error rate/oversights in detecting abnormalities and the potential inter- and intra-observer variability [25].

The value and reliability of a SB-CE are initially dependent on the bowel cleanliness achieved in the preparation for the procedure, which, if inadequate, undermines the reliability of any failure to detect abnormalities. Thus, it is essential to have a reliable, objective, and reproducible scoring tool to assess the quality of SB preparation in CE that can be used in conjunction with any manual or automatic detection system. This need has prompted the development of AI algorithms to automatically assess SB cleanliness during CE [21,26,27,28,29]. This study set out to design and develop a CNN that could be used in an AI system to automatically classify bowel preparation in CE images and to validate the performance of this tool on a large set of real-world CE images.

## 2. Materials and Methods

### 2.1. Study Design

The CE images obtained from the examination of patients (*n* = 4319) between 2015 and 2022 at the São João University Hospital (Porto, Portugal) and the Manoph Gastroenterology Clinic (Porto, Portugal) were reviewed retrospectively in this study. These patients were referred for CE examinations as a result of detecting indications of a small-bowel disorder or for the monitoring of any confirmed small-bowel diagnosis. The complete video of the examinations performed was reviewed, extracting 12,950 images of the SB mucosa in total. A total of 3 experienced gastroenterologists (M.M.S., H.C. and A.P.A), experts in SB-CE who had each reviewed over 1500 CE examinations before the start of this study, carried out the inclusion and labeling of frames. The three experts in CE analyzed the still frames independently and scored the quality of SB cleansing based on the proportion of mucosa visualized and in accordance with the degree of obscuration by bubbles, bile and/or debris. The images were selected from an ever-growing CE images database. Accordingly, the quality of SB cleansing in each still frame was categorized as excellent (E) when ≥90% of the mucosa was visible, satisfactory (S) when 50% to 90% of mucosa was visible, and unsatisfactory (U) when <50% of the mucosa was visible. This classification was irrespective of the presence or absence of any endoscopic lesions. The final labeling of each frame required the agreement of all three experts regarding the quality of bowel preparation. Frames in which a consensus was not reached were not included in the analysis to ensure that the frames studied were correctly labelled.

The ethics committee at São João University Hospital/Faculty of Medicine of the University of Porto (No. CE 407/2020) approved this study, which was carried out in accordance with the Helsinki declaration guidelines for research on human subjects. In this non-interventional retrospective study, no information that might potentially identify any of the subjects was presented, assigning every patient a randomly generated numeric code to ensure data anonymization in compliance with the general data protection regulation (GDPR). The data was confirmed to be non-traceable by experts with Data Protection Officer (DPO) certification (Maastricht University).

### 2.2. SB-CE Procedure

A total of 5793 SB-CE exams were included. Two SB-CE systems were used during the study period: the PillCam SB3 (*n* = 4509, Medtronic, Minneapolis, MN, USA) or the OMOM HD (*n* = 1284, Jinshan Science & Technology Co., Chongqing, China). Both of these systems rely on an endoscopy capsule and a sensor that connects to a data recorder, accompanied by specific software to review the images. The Pillcam SB3 capsule is 26.2 mm long and 11.4 mm wide, with ≥8 h of estimated battery life, and its high-resolution camera has a wide field of view (156°). The rate of capture varies between 2 and 6 frames per second (fps), shifting automatically in response to the speed at which the capsule progresses, and the images are analyzed with the PillCam Software version 9 (Medtronic). Conversely, the OMOM HD capsule is slightly longer (27.9 mm) and wider (13.0 mm), with a 172° field of view. The adaptive frame rate of this system is from 2 to 10 fps and the Vue Smart Software (Jinshan Science & Technology Co.) is used to review the images from this capsule. All the images obtained were processed to ensure that potential identifying information (name, ID number, date of the procedure, etc.), and each frame extracted was numbered consecutively prior to storage.

Each patient followed a protocol for bowel preparation that was in accordance with the European Society of Gastrointestinal Endoscopy (ESGE) guidelines [11]. The day prior to capsule ingestion patients followed a clear liquid diet, fasting overnight before the examination. They drank a polyethylene glycol (PEG) solution (2 L) prior to ingesting the capsule, that included simethicone to prevent foaming. After ingestion of the capsule, the patients were not allowed to eat for 4 h, and if the capsule had not been expulsed 1 h after ingestion and it remained in the stomach, prokinetic therapy was applied (10 mg of domperidone) with the ESGE recommendations [11].

### 2.3. Development of the CNN

**Figure 1:**
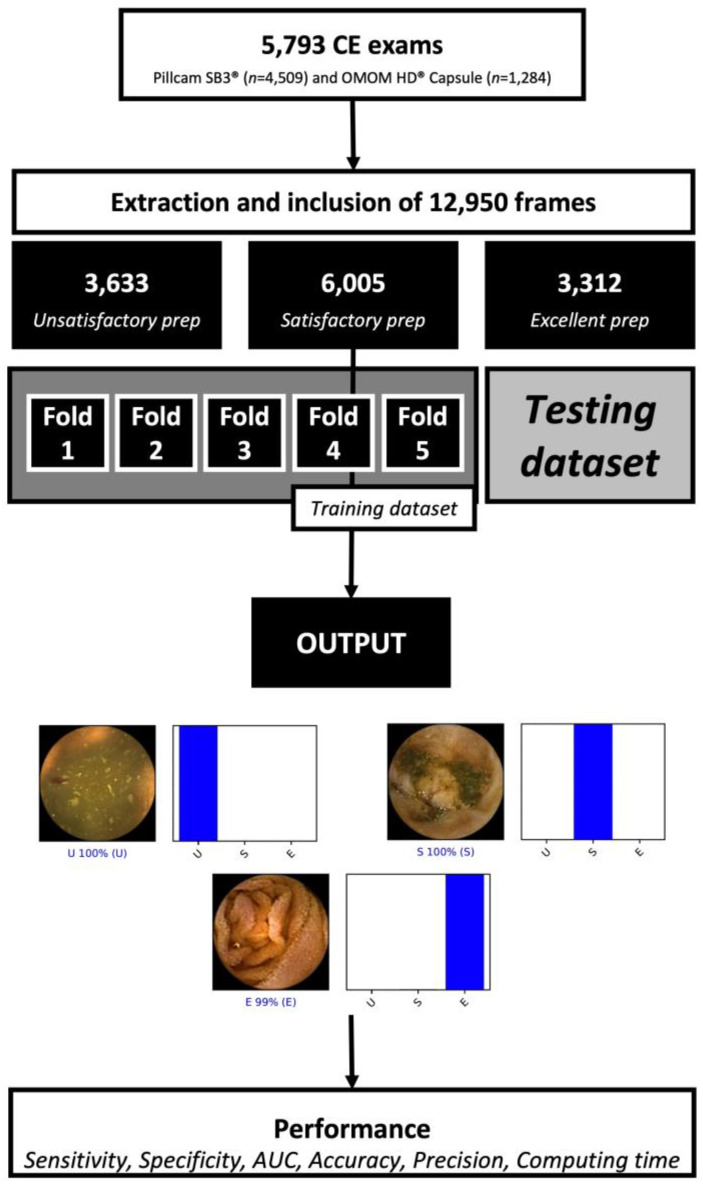
Flowchart indicating the procedures carried out in the training phase and the validation phase of the study, and indicating the proportion of the examinations carried out with each type of capsule. A five-fold cross validation examination was performed in the training stage. The level of cleanliness (Output) was classified as: Excellent, excellent bowel preparation (≥90% of the mucosa visualized); Satisfactory, satisfactory bowel preparation (50–90% of the mucosa visualized); Unsatisfactory, unsatisfactory bowel preparation (<50% of the mucosa visualized). Abbreviations: CE, Capsule Endoscopy; PPV, positive predictive value; NPV, negative predictive value; AUC; area under the ROC curve.

The deep learning algorithm was designed to provide the automatic classification of small-bowel preparation according to the aforementioned categories. A total of 12,950 images were included (*n* = 9983 from the PillCam SB3 system and *n* = 2967 from the OMOM HD system). The total dataset was divided for constitution of training and testing sets, according to a patient-split approach, therefore ensuring that data from the same patient were not present in the training and test datasets simultaneously. The flowchart below summarizes how the study was performed and how the images to train and validate the CNN were selected (Figure 1).

During the training phase, we performed a 5-fold cross validation. Within each fold, the total training set was randomly divided into 5 even-sized groups. Within each fold, 4 groups were used for training (80%) and 1 group for testing (20%). The distribution of the groups varied between each fold. The specifications of the model were tuned for each run. The specifications applied to the best performing fold were applied during the test stage. The CNN was generated based on the RegnetY model and trained using ImageNet, a large image dataset used commonly when developing object recognition software. The model’s convolutional layers were retained to pass on this learning to our data, although the last fully connected layers were removed and attaching fully connected layers in accordance with the number of classes used to evaluate the CE images. Two blocks were used that each had a fully connected layer followed by a dropout layer with a 0.3 drop rate. A dense layer was added after these two blocks, defining its size as the number of categories in the classification (three). The learning rate (0.00025), batch size (128) and epoch number (10) were established through trial and error. The data was prepared with the PyTorch 1.11 library, which were also used to run the model, and these analyses were carried out using a computer with a 2.1 GHz Intel Xeon Gold 6130 processor (Intel, Santa Clara, CA, USA) and a dual NVIDIA Quadro RTX A6000 graphics card (NVIDIA Corporate, Santa Clara, CA, USA).

### 2.4. Model Performance and Statistical Analysis

The primary outcome measures included sensitivity, specificity, positive predictive value (PPV) and negative predictive value (NPV), and overall accuracy. Moreover, we analyzed the receiver operating characteristic (ROC) and area under the ROC (AUC) curves to assess how well the model could distinguish the three categories. The classification predicted by the CNN was compared to that achieved by expert consensus, considered to be the gold standard. Furthermore, the network’s capacity to process images was evaluated by quantifying the time the CNN required to reach a classification for all the validation images in the test dataset.

**Figure 2:**
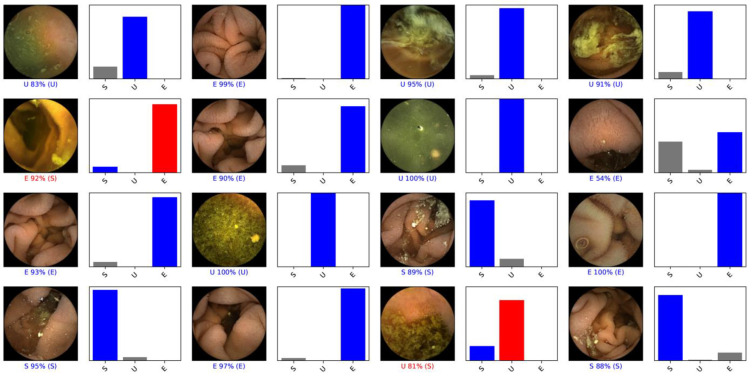
Output obtained by implementing the CNN. The bars represent the probability estimated by the network and the finding with the highest probability was considered to be the predicted classification output. The blue bar represents a correct prediction: E, excellent bowel preparation (≥90% of the mucosa visualized); S, satisfactory bowel preparation (50–90% of the mucosa visualized); U, unsatisfactory bowel preparation (<50% of the mucosa visualized).

The probability that the trained CNN would attribute each of the three categories to an image (excellent, satisfactory or unsatisfactory) was calculated. The higher the probability, the greater the confidence in the CNN prediction, such that the category carrying the highest probability score was considered as the classification output predicted by the CNN (Figure 2). The sensitivity, specificity, accuracy, PPV and NPV to differentiate the three small-bowel preparation classes was calculated. Receiver operating characteristic curves (ROC) and the area under these curves (AUC) were used to assess the performance of the CNN to detect and differentiate the different SB preparation classes. Statistical analyses were carried out with Sci-Kit learn v.22.2 software [30].

## 3. Results

### 3.1. Convolutional Neural Network Construction and Training

In total, 12,950 SB-CE images were used for the construction of the neural network. Of the 5793 procedures undertaken, 4509 were carried out using the Pillcam SB3 capsule (Medtronic), while the OMOM HD capsule (Jinshan Science and Technology) was used in 1284 examinations. From the included cohort of images, 3633 were labeled by the experts as unsatisfactory preparation, 6005 as satisfactory preparation and 3312 as excellent preparation. From the total dataset, 94% of the images (*n* = 12,159) were used during the training stage and 6% (*n* = 791) were reserved for independent testing of the model.

During the training stage, a five-fold cross validation was performed. The results for each of the folds are presented in Table 1. During the training stage, the model achieved a mean sensitivity of 88.4% (CI 95% 83.8–93.0%), specificity 93.6% (CI 95% 90.2–96.9%), and accuracy 92.1% (CI 95% 89.5–94.6%)).

### 3.2. Global Performance of the CNN to Differentiate the Classed of Small-Bowel Cleanliness during the Testing Phase

**Figure 3:**
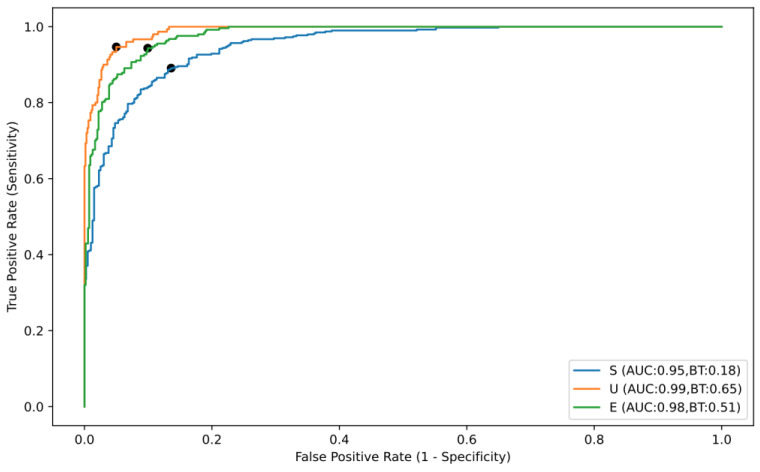
Receiver operating characteristic (ROC) curve of the convolutional neural network’s performance in differentiating the small-bowel preparation classes: AUC, area under the ROC curve. (BT—best threshold).

The level of performance of the CNN was assessed based on the AUC, accuracy, sensitivity, specificity, PPV and NPV. Overall, the deep learning algorithm proved to be capable of automatically differentiating small-bowel preparation classes with a calculated accuracy of 89.1%, a sensitivity of 87.6%, and a specificity of 92.2% (Table 2). The individual performance marks for each of the categories are shown in Table 2. The ROC analyses and respective AUCs (Figure 3) indicated the performance of the CNN in differentiating excellent, satisfactory, and unsatisfactory cleanliness in SB preparations was high, with AUCs of 0.98, 0.95 and 0.99, respectively.

### 3.3. Computational Performance of the CNN

At the best-performing fold during the training stage, the CNN completed the reading of 790 batches of 128 images in 198 s, which corresponds to a reading rate of approximately 504 frames per second. If this performance is applied to a complete CE examination, the video of which contains approximately 50,000 frames, an estimated 99 s would be the time required for its full analysis.

## 4. Discussion

In this study, we present a novel AI tool that adopts a multi-layered CNN designed to automatically assess the degree of bowel cleanliness in images obtained from CE examinations. Following its training on a large dataset of real-world images, the capacity of the CNN to establish bowel cleanliness was tested on a large validation dataset, demonstrating a very high level of accuracy, sensitivity and specificity relative to the current gold standard. Consequently, we believe that this tool represents an interesting advance in the search for AI tools that can enhance the yield and efficacy of CE procedures, which is worthy of further study.

CE is becoming an important technique to study small-bowel disorders, yet despite the improvements it can offer (such as in image quality and the localization of the capsule or lesions), this technique is still subject to the time constraints and effort required for reading by a gastroenterologist. This monotonous and time-consuming task is unfortunately associated with poor reproducibility and consequently, possible failure in detecting lesions or abnormalities that may be small and present in only a few frames. Thus, AI tools are being designed to automate the reading process and to detect and/or analyze gastrointestinal lesions [21,23,24,31,32,33,34,35,36,37,38], with the aim of alleviating the burden on gastroenterologists associated with manual reading, and reducing reading time without compromising accuracy.

In order to ensure the successful detection of abnormalities in CE examinations, it is essential to achieve adequate bowel preparation [6,11,12,13,39,40]. Despite the importance of adequate bowel preparation to ensure CE examinations are conclusive, and the range of cleanliness grading scales available for SB-CE with very different technical characteristics [15], there is as yet no consensus on an objective and reliable scoring system to assess SB cleanliness following CE preparation. Moreover, there is still no agreement on the most appropriate protocol for the preparation of CE examinations [6,14,41,42,43,44], although there is evidence that the use of PEG/ascorbic acid booster following a standard preparation enhances mucosal visualization [43,45]. Thus, an additional tool that will be fundamental in the drive to automate the evaluation of CE examinations is a system to evaluate the cleanliness of the GI tract through the images extracted. Indeed, small-bowel cleanliness will become more important in the future to ensure that the AI applications designed to evaluate the small-bowel mucosa using deep learning models can achieve excellent diagnostic yields.

The deep learning tool designed here to automatically differentiate the cleanliness of the small bowel in CE images addresses this important issue. The CNN model tested was trained using a large dataset of 12,950 real-world images in order to enhance its accuracy. Importantly, all the images used had been classified in the same way by three experts with large experience in CE (>1500 CE exams prior to this study), ensuring there was no ambiguity in their status as well as the accuracy of the CNN. Indeed, the larger the number and variety of images used to train an algorithm, the more efficient it will be, more closely reflecting circumstances encountered in clinical practice. Moreover, the performance of this algorithm was assessed using strict patient-split rules, ensuring that there was no overlap of data between training and testing dataset. The concomitant performance of a five-fold cross validation further reduces the risk of overfitting of the model, strengthening the robustness of the model and validity of the results. This CNN was tested using an independent set, demonstrating high levels of performance in differentiating different levels of SB preparation according to a simplified three-level classification scale of cleanliness that is based on the proportion of the SB mucosa that can be visualized in each image. Testing the CNN revealed an accuracy of 89.1%, a sensitivity of 87.6% and a specificity of 92.2% relative to the gold standard. In addition, the AUCs to differentiate the different categories of SB preparation quality varied between 0.95 and 0.99. In terms of the image processing performance, the CNN used here read the validation data at a frame rate of 504 fps, which would ensure that a complete CE video that generally contains around 50,000 frames could be examined in under 2 min.

The performance of the CNN presented here is similar or superior to those of recent applications exploring CNN architectures for automatic assessment of cleanliness in CE examinations. For example, an accuracy of 95.2%, a sensitivity of 96.2% and a specificity of 94.3 were reported recently when classifying images into four cleanliness categories according to the presence of intestinal content [27]. The CNN model used for this was trained with a large number of images (*n* = 55,293) but tested on a more limited number of images (*n* = 854) from 30 new CE videos collected in a clinical setting. A neural-network-based algorithm was also developed subsequently and trained with only 600 SB images, categorizing their cleanliness as adequate or inadequate according to a 10-point scale [26]. The validation of this algorithm reflected a sensitivity of 90.3%, a specificity of 83.3% and an accuracy of 89.7%, although this was based on the use of only 156 SB-CE video recordings. However, the more extended use of this algorithm and the ensuing learning undertaken may enhance its performance. More recently, a deep-learning-based algorithm was developed in a preliminary study trained with 71,191 images [28] and it was used to design software to evaluate SB preparation quality classified according to a five-point scoring system that evaluated the clarity of mucosal visualization. This tool was trained on a very large number of images 280,000 and verified on 120,000 images [29], and the performance of the algorithm provided an accuracy of 93%. ROC curve analysis using an external validation set of 50 CE cases separate from the training set defined a sensitivity of 81%, a specificity of 84% and an AUC of 0.913, again below the numbers achieved here.

While there are currently few studies aimed at automatically assessing the cleanliness of CE bowel preparations using deep learning applications, there are several important aspects of this study that should be emphasized, as well as certain limitations. In the first place, it is noteworthy that this CNN was applied to two different CE systems (Pillcam SB3 and OMOM HD), with different specifications and optical performance. Moreover, while this was not an extensive multicenter study, the images used were recorded at two centers (albeit in the same city), suggesting that it may be reproducible in different centers. However, further studies will be necessary to ensure that this application can be implemented on a more universal basis. Another important highlight is that this CNN was designed using a large patient and image dataset, using a patient-split approach, which assured that there was no overlap of patients between the training and testing sets. Moreover, we applied a five-fold cross validation which further strengthens the methodological robustness of the model.

This study has several limitations. First, although this study offers evidence that the CNN may be widely applicable to different systems, we did not assess the performance in the validation dataset with either system individually, which would be of interest to determine if there might be differences with distinct systems that would affect the generalization of our model to other CE systems. Second, this study was conducted in a retrospective fashion and, although the dataset is relatively large, further developments in the range of AI for capsule endoscopy will require the performance of larger multicenter studies evaluating the performance, validity and reproducibility of the CNN in a real-life setting. Third, the CNN was developed by analyzing still frames and, thus, it will be crucial to assess how this model performs when using full-length videos before it can be integrated into CE reading systems in clinical practice. Fourth, the categories were defined with optical revision by three reviewers and no image segmentation tool was used to define the percentage of the frame showing visible mucosa more objectively. This limitation may explain the relatively poorer accuracy of the CNN in distinguishing between satisfactory and excellent or unsatisfactory bowel preparations. Fifth, subsequent studies should assess the impact of capsule findings in the bowel preparation classification by the CNN. Finally, it will be important to determine how the CNN performs when different protocols of bowel preparation are followed, not only to assess the performance in relation to the different preparations used for CE, but also to potentially help adopt the most appropriate strategy to optimize the cleanliness for CE examination.

Ideally, AI algorithms for the automatic classification of small-bowel preparation should be integrated into CE reading tools together with AI algorithms for the automatic differentiation of images with a normal or abnormal mucosa. This will allow images with normal mucosa and images with poor cleanliness quality to be filtered out, enabling the gastroenterologist to focus on suspected lesions. Consequently, this will improve the diagnostic yield and lessen the burden on the gastroenterologist in terms of time and effort, while also reducing the associated costs.

## 5. Conclusions

A CNN-based model was developed to automatically classify bowel preparation for CE examinations based on a simple quantitative scale. The implementation of systems that automatically assess bowel cleanliness in CE is likely to enhance the reliability and reproducibility of the scales used to evaluate bowel preparation, and the performance of tools to detect GI tract or small-bowel lesions.

## Figures and Tables

**Table 1:** Five-fold cross validation during the training phase.

	Sensitivity (%)	Specificity (%)	PPV (%)	NPV (%)	Accuracy (%)
Fold 1	88.8	93.4	86.2	92.8	91.5
Fold 2	92.8	95.9	93.9	94.5	94.8
Fold 3	90.1	95.3	88.7	95.1	93.4
Fold 4	91.2	94.8	90.7	94.7	93.6
Fold 5	79.3	88.4	83.0	89.7	87.1
Overall mean (CI 95%)	88.4 (83.8–93.0)	93.6 (90.2–96.9)	88.5 (84.9–92.1)	93.4 (90.6–96.1)	92.1 (89.5–94.6)

Abbreviations: PPV—positive predictive value; NPV—negative predictive value; CI 95%–95% confidence interval.

**Table 2:** CNN performance for detection and differentiation of small-bowel preparation categories.

	Sensitivity	Specificity	Accuracy
Overall, %	87.7	92.2	89.1
U vs. all, %	96.7	91.7	92.7
S vs. all, %	72.1	95.2	83.7
E vs. all, %	94.3	89.5	91.0
E vs. S, %	94.3	83.2	87.9
E vs. U, %	100.0	100.0	100.0
S vs. U, %	84.3	96.7	88.1

Abbreviations: CNN—convolutional neural network; U—unsatisfactory; S—satisfactory; E—excellent. SD—standard deviation.

## Data Availability

Not applicable.
